# Concerning Predation on Small Vertebrates by Alien Gold Morph Midas cichlid, *Amphilophus citrinellus* (Cichlidae)

**DOI:** 10.21315/tlsr2024.35.2.15

**Published:** 2024-07-31

**Authors:** Mohamad Aqmal-Naser, Amirrudin B. Ahmad

**Affiliations:** 1 Terrestrial Ecology, Biodiversity and Aquatic Research (TEBAR), Institute of Tropical Biodiversity and Sustainable Development, Universiti Malaysia Terengganu, 21030 Kuala Nerus, Terengganu, Malaysia; 2 Biodiversity and Ecology Research (BERes), Faculty of Science and Marine Environment, Universiti Malaysia Terengganu, 21030 Kuala Nerus, Terengganu, Malaysia

**Keywords:** Biodiversity, Introduced Species, Invasiveness, Peninsular Malaysia, Rice Agro-Ecosystem, Biodiversiti, Spesies Pengenalan, Invasif, Semenanjung Malaysia, Agro-Ekosistem Beras

## Abstract

Neotropical cichlid possesses territorial aggression which explains their success as alien species that pose threats to local fauna. The feeding ecology of Midas cichlid, *Amphilophus citrinellus* species outside its native range had never been fully understood. We aim to determine the stomach content, length-weight relationship and condition factor of this non-native species in one of the agroecosystems in Malaysia. The fish was collected using a cast net, and the guts were dissected. The stomach content (*n* = 35) revealed Midas cichlids feed on a wide array of preys including fish, amphibian and gastropod. The *b*-value is 2.60 (negative allometric growth) and the relative condition factor, Kn is 1.04. This result represents an initial study on the feeding aspect of this cichlid. Subsequent and continued researches are needed to evaluate the feeding behaviour and prey preferences of this species in its introduced range.

## Abstrak

Cichlid neotropika mempunyai pencerobohan wilayah yang menjelaskan kejayaan mereka sebagai spesies asing yang menimbulkan ancaman kepada fauna tempatan. Ekologi pemakanan Midas cichlid, spesies *Amphilophus citrinellus* di luar julat asalnya tidak pernah difahami sepenuhnya. Kami berhasrat untuk menentukan kandungan perut, hubungan panjang-berat dan faktor keadaan spesies bukan asli ini dalam salah satu agroekosistem di Malaysia. Ikan dikumpul menggunakan jaring tuang dan ususnya dibedah. Kandungan perut (*n* = 35) mendedahkan Midas cichlids memakan pelbagai jenis mangsa termasuk ikan, amfibia dan gastropod. Nilai *b* ialah 2.60 (pertumbuhan alometrik negatif) dan faktor keadaan relatif, Kn ialah 1.04. Keputusan ini mewakili kajian awal tentang aspek pemakanan cichlid ini. Penyelidikan seterusnya dan berterusan diperlukan untuk menilai tingkah laku pemakanan dan keutamaan mangsa spesies ini dalam julat yang diperkenalkan.

**Highlights**

- Midas cichlid feed on a wide array of preys including fish, amphibian and gastropod.
- Midas cichlid in the rice agroecosystem have negative allometric growth(*b* = 2.60) and the relative condition factor, Kn is 1.04.
- New information on the feeding ecology of Midas cichlid in their introduced range.

## INTRODUCTION

Midas cichlid, *Amphilopus citrinellus* exhibits polychromatism, with two general colour morphs throughout its growth induced by breeding mode, location and water depth (Barlow 1976). The gold morphs *A. citrinellus* were typically aggressive, possibly as a response to predation (Barlow & Wallach 1976), and also owing competition over limited resources in their niche (Oldfield 2011), allowing them to survive outside the native range. This morph also showing the domination against the normal morph in term of predation and growth (Barlow *et al*. 1975). We believe such territorial aggression may be demonstrated constantly by *A. citrinellus* gold morph fish and it is persisting even when found outside their native environment.

*A.citrinellus* has been reported from outside its native ranges including Australia (Kroon *et al*. 2015), Indonesia (Tampubolon *et al*. 2015), Malaysia (Aqmal-Naser & Ahmad 2018a; 2020), Philippines (Poniente *et al*. 2019) and Singapore (Tan *et al*. 2020). Established feral populations of *A. citrinellus* have been reported from several countries including Singapore (Kwik *et al*. 2013) and Indonesia (Tampubolon *et al*. 2015), which suggest that this species soon could be another cichlid species invading natural waterbodies after tilapias (*Oreochromis* spp.). However, the feeding ecology of *A. citrinellus* outside its native range, especially in Peninsular Malaysia, is relatively unknown.

In this study, we examined the stomach contents of *A. citrinellus* populations thriving in agro ecosystem to investigate the potential impacts of this alien fish species to native biota. Since food is one of the keys in determine fish condition, population, level and growth rate (Begum *et al*. 2008), the information gathered on the diet of fish is crucial to understand their behaviour, biology and physiology (Shalloof *et al*. 2020). The study on feeding habit also can help to explain the biology of single species (Graham *et al*. 2007), and the trophic flow of the ecosystem (Cox *et al*. 2023). Our study is important for establishing the fundamental criteria of the potential invasiveness of the aggressive *A. citrinellus* in Peninsular Malaysia and the findings could be applicable to other places outside its native distribution range.

## MATERIALS AND METHODS

The occurrence of *A. citrinellus* at this study sites has been previously reported by Aqmal-Naser and Ahmad (2020). The samples of *A. citrinellus* were collected opportunistically from the rice field. Samples were collected using cast net with a dimension of 2.4 m height × 10 m circumference × 2 cm mesh size. All collected specimens were measured for standard length (SL; tip of the upper jaw to the base of caudal fin to the nearest mm). The gut was removed and dissected for visual examination of the stomach contents and the occurrence of the eggs in the abdominal cavity was recorded to indicate their ability to reproduce and to establish feral population at the study area. Any larger native fauna found in the stomach were identified using visible characters to the lowest possible taxon. The identification of fish species was based on Zakaria-Ismail *et al*. (2019), while the identification for frog and toad species were based on Grismer (2011). The fish stomach content analysis was done using Occurrence Method (Frequency of occurrence; %) as suggested by Hyslop (1980). The number of stomachs contain one or more individuals for each category of food and expressed as percentage (Kennedy & Fitzmaurice 1972). All specimens were preserved in 10% formalin solution and transferred to 75% ethanol after two weeks and deposited in Universiti Malaysia Terengganu Zoological Collection (UMTZC). The growth condition (*b*-value) of the fish were determined following the formula *W* = *aL^b^* (Ricker 1973) where *W* = weight of fish in gram; *L* = Length of fish in cm; *a* = describe the rate of change of weight with length (intercept); and *b* = weight at unit length (slope), while relative condition factor (Kn) following Le Cren (1951).

## RESULTS

**Figure 1:**
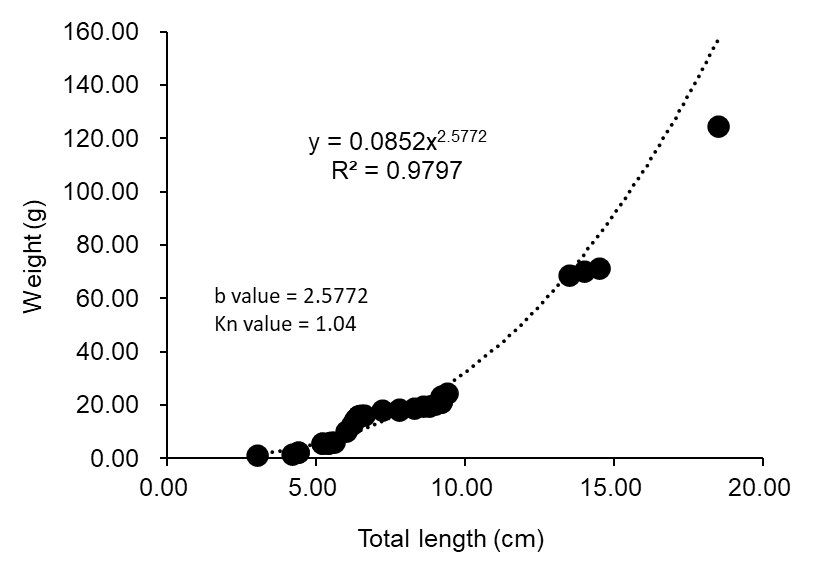
Length-weight relationship of Midas cichlid in the study site (*n* = 35).

**Figure 2:**
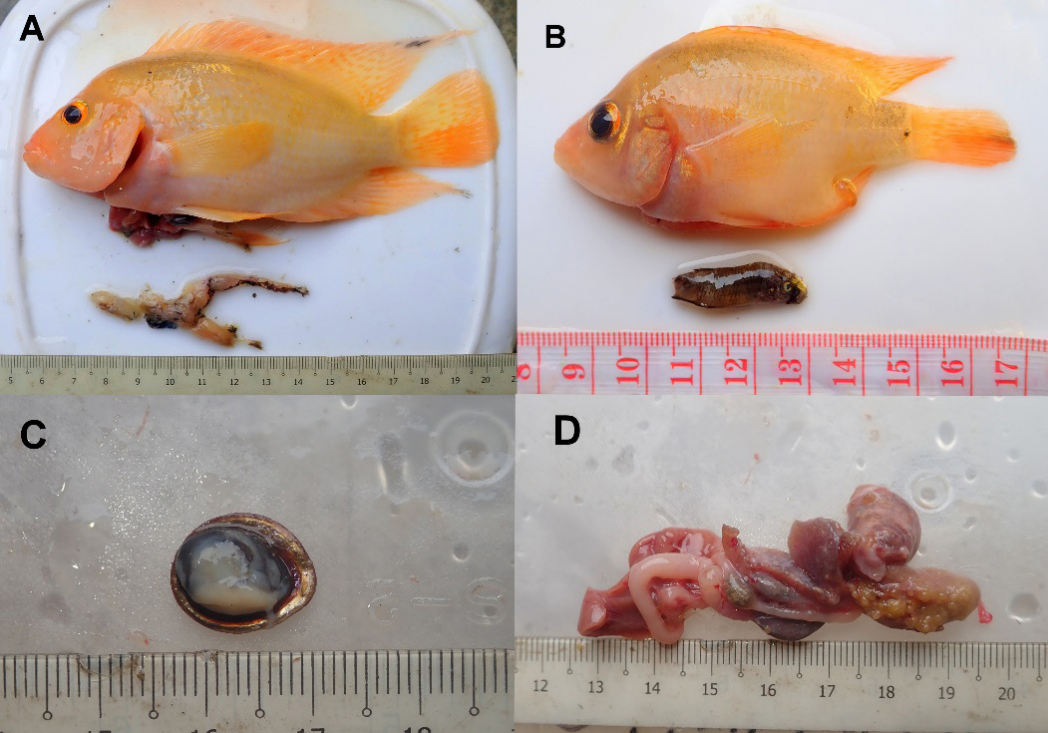
The stomach contents of *Amphilophus citrinellus* containing: (A) partially digested native frog (Dicroglossidae); (B) native fish, *Betta imbellis*; (C) the operculum size of the gastropod; and (D) the remnant of gastropod along with egg cluster.

From the survey, we collected 35 specimens of gold morphs *A. citrinellus* (total length; 3.00 cm–18.50 cm, and weight 1.30 g–254.22 g). The growth condition (*b*-value) for the cichlid is 2.60, while the relative condition factor (Kn) is 1.04 (Fig. 1). The stomach content from two individuals contained native fauna; frog (Family Dicroglossidae), and a native fighting fish species (*Betta imbellis*). One individual is a gravid female and the stomach contain the remain of unidentified gastropod (Fig. 2). Stomach content of 20 individuals contain unidentified fish scales while the other 12 individuals’ fish had empty stomachs.[Table t1-tlsr-35-2-309]

## DISCUSSION

Documented evidence of such predation by alien fishes in Peninsular Malaysia is scarce, with the exception of *Clarias gariepinus* (Aqmal-Naser & Ahmad 2019). Several small-bodied native fish species had been previously reported at the study site (Aqmal-Naser & Ahmad 2018b), where some of these native species hold commercial potential for small-scale ornamental trade [(e.g., *Rasbora trilineata*, *Oryzias javanicus, Lepidochepalichthys hasselti* and *Betta imbellis* (Aqmal-Naser & Ahmad 2018b)], but may be threatened by the predation pressure from *A.citrinellus*. Given the aggressive behaviour demonstrated by this speciescomplex, the gold morph of *A. citrinellus* has been known to attack and eat small fishes (Barlow *et al*. 1975). Aggression behaviour demonstrated by this Midascichlid may be extended to the feeding needs of the fish. The fish not only respond aggressively towards their own morph as previously reported (Lehtonen 2014)but may aggressively prey upon the native species. Our study presents the first evidence on the predation of native frog and fish species, and also confirming the predation on snail species by *A. citrinellus* in Peninsular Malaysia. Since *A.citrinellus* has now occurred regularly in human-modified habitats (Kwik *et al*. 2013; Tampubolon *et al*. 2015), impacts on native fish community may be amplified in the near future if precautionary steps are not taken.

Several other predatory fish species can be found in the rice field (see Aqmal-Naser & Ahmad 2018a; 2018b). Many of these fishes feed on invertebrates, small fishes, and a few species such and the snakehead, *Channa striata*, the top predator in this agro ecosystem, feed on vertebrates including frogs. Dicroglossid frogs are common and widespread in the rice fields in Peninsular Malaysia (Shahriza & Ibrahim 2017) and are preyed by many species including snakes. With that being said, predation of a native frog by an alien fish has not been previously reported. The unexpected finding of *A. citrinellus* feeding on a native dicroglossid frog is recorded for the first time in Peninsular Malaysia. Elsewhere, predation of native frogs by the alien fish species, *Clarias gariepinus* has been previously documented in Cuba (Rodríguez-Machado & Rodríguez-Cabrera 2015), even on the critically endangered spiny-chest frog (*Alsodes pehuenche*) by introduced rainbow trout, *Onchorhynchus milkyss* (Zarco *et al*. 2020).

Our finding of *A. citrinellus* feeding on the native frog not only represents a threat to native fauna, but also may also imply competition with native predators for the same food resources. Without appropriate control measures, this may contribute to the loss of native biodiversity. Eventually, long term effects of alien fish species in the natural and man-made habitats are proliferated. Thus, a systematic and urgent plan is required for eradicating the invasive species plus further study and continued monitoring of this event must be executed immediately. Both *A. citrinellus* (in this study) and *Clarias gariepinus* (of the previous study) were recorded feeding on the native species within the same study area. Upon reaching a certain size, both of these alien species have no native predators (with the possible exception of the water monitor lizard, *Varanus salvator*). Given that this man-made ecosystem contains many small-bodied fish species, predation on native species likely occurs more frequently. Since both alien fish species feed on the same resources, competition for food between *A. citrinellus, C. gariepinus* and the native *Channa striata* may finally intensify.

While there is currently limited evidence of breeding populations (e.g., lack of juveniles), their feeding preferences posed a potential threat to the native biota. The initial discovery on a pair of *A. citrinellus* which the female is gravid (Fig. 2) could affirmed the possibility of establishing the breeding population soon. In the man-made habitat in Singapore, the species was known to have established population based on the size range from small to large and the presence of breeding pit (Kwik *et al*. 2013). Furthermore, Purnamaningtyas and Tjahjo (2017) reported that *A. citrinellus* has high fecundity in the reservoir and can breed throughout the year given that the water temperature ranging from 24°C–28°C.

Purnamaningtyas and Tjahjo (2017) also reported larger Midas cichlid has higher fecundity, where each female has about 1,000–3,000 eggs. They usually lay their eggs on the ceiling of natural caves or hard substrates (Lavery 1991), which in this study, the pair was collected from the concrete canals. However, the reproductive aspect of Midas cichlid is complex. The species is monogamous and spawning selection is complex (Rogers 1987). The female tends to choose normal and primitive colour male, the largest and the most aggressive male to spawn (Barlow 1992). Increasing number of male or crowding effect will have influence on the mating of Midas cichlid (Rogers 1987). The male is not choosy, but female will usually have individual selection, where it will choose male with high tendency to be a good father to the fry (Barlow 1992).

Midas cichlid population in this study shows negative allometric growth (*b* < 3.0) where the length increases but the weight did not increase proportionally. The relative condition factor of 1.04 also shows that the Midas cichlid population having the borderline growth condition between poor and good. The fish become slimmer or thinner, as one of the reasons, is the lack of food resources (Jisr *et al*. 2018). The empty stomach of 34.29% indicated the scarcity of food availability, hence Midas cichlid could devour any organism available including the frog. Plus, this agroecosystem is a highly disturbed environment, both temporally and spatially (Aqmal-Naser *et al*. 2023). Such factors as habitat and environments, are among the major factor contributing to the poor growth of fish (Morato *et al*. 2001). Compared to the related study, Midas cichlid in Indonesia has isometric growth (*b* = 3.5), as they consumed diverse food including insect larvae, insect and plant matters (Purnamaningtyas & Tjahjo 2017).

We were aware that the sample size of this cichlid in this study can be considered small (*n* = 35 individuals). Nevertheless, the veracious feeding behaviour shown through this observation is a clear indication that once the population of this alien species established, similar feeding behaviour will be observed and become more prominent, especially when the competition for food is escalating. Our finding provides an impetus for further studies on the potential impacts by *A. citrinellus* in other human-modified habitats, particularly in the urban areas of the central west coast (i.e., Selangor) and southern Peninsular Malaysia (e.g., Johor and Negeri Sembilan), which man-made habitats such as retention ponds, reservoirs and ex-mining pools are abundant that *A. citrinellus* was thriving well. Additional sampling on the other states in Peninsular Malaysia may provide ancillary data for better understanding on the impacts of this fish to the native biota. This data then can be used to suggest and provide crucial information on the potential “hotspots” of alien fish species in Peninsular Malaysia, which is important for informing potential control and/or extermination measures. Another future studies should explore on the possibility of *A. citrinellus* demonstrate bias aggression towards their own morph as this might explain the less numerous individuals collected during the sampling.

The occurrence of alien fish species with potential to become invasive in man-made and natural water bodies urgently requires monitoring and control. Owing to the limited information on their feeding ecology and biology, alien fish species were not previously regarded as a threat to native fauna and have hitherto of less interest from the researchers in Peninsular Malaysia. The concrete eradication measures are urgently needed as the negative ecological impacts of alien species start to escalate.

## Figures and Tables

**Table 1 t1-tlsr-35-2-309:** The stomach content analysis for *Amphilophus citrinellus* (*n* = 35) with the percentage of occurrence (%FO) of three groups of fauna.

Class	Family	(% FO)	Remarks
Actinopterygii	Opshronemidae	2.86	*Betta imbellis* (*n* = 1)
	Unidentified species	57.14	Unidentified fish scales (*n* = 20)
Amphibia	Dicroglossidae	2.86	Partially digested frog (*n* = 1)
Gastropoda	Ampullariidae	2.86	Unidentified mollusc (*n* = 1)
Empty stomach	-	34.29	*n* = 12
